# Identification of human parvovirus 4 genotypes 1 and 2 in Chinese source plasma pools

**DOI:** 10.1002/jmv.26666

**Published:** 2021-03-14

**Authors:** Junting Jia, Yadi Zhong, Huan Zhang, Dian Yuan, Limin Ma, Deqing Wang, Jingang Zhang, Yuyuan Ma

**Affiliations:** ^1^ Department of Transfusion Medicine The First Medical Center, Chinese PLA General Hospital Beijing China; ^2^ NMPA Key Laboratory for Quality Control of Blood Products Institute of Health Service and Transfusion Medicine, Academy of Military Medical Sciences Beijing China; ^3^ Department of Hematology Second Medical Center of Chinese PLA General Hospital Beijing China

**Keywords:** genotype, human parvovirus 4, human parvovirus B19, quantitative PCR, source plasma pools

## Abstract

Human parvovirus B19 (B19V) and human parvovirus 4 (PARV4) are known to infect humans and transmit through contaminated blood and blood products. Globally, three genotypes of B19V, as well as PARV4, have been identified, respectively. The existence of different B19V genotypes in Chinese plasma donors has been investigated, however, the data regarding PARV4 were not available. The main objective of this study is to identify the genotypes of PARV4 circulating in Chinese plasma donors. By using a duplex quantitative polymerase chain reaction assay adapted for all genotypes of B19V and PARV4, 78 source plasma pools for fractionation were screened and quantified. Results showed that positive rates of B19V and PARV4 DNA in plasma pool samples were 25.64% and 14.10%, respectively. PARV4 sequences in two positive samples were next genotyped, and these two sequences belonged to PARV4 genotypes 1 and 2, respectively. In conclusion, the data present demonstrate the existence of PARV4 genotypes 1 and 2 in Chinese plasma donors for the first time and also show the relatively lower prevalence and level of PARV4 DNA in Chinese plasma donors in comparison with that of B19V DNA.

## Highlights

At least two PARV4 genotypes, 1 and 2, were currently present in China.

The prevalence and level of PARV4 DNA in Chinese plasma donors were relatively lower in comparison with that of B19V DNA.

The rate of B19V and PARV4 coinfection in Chinese source plasma pools was low.

## 1 INTRODUCTION

Blood products refer to therapeutic products derived from human blood or plasma, such as human albumin, human immunoglobulin, and human coagulation factors. Viral safety of the products is the top priority of the blood products industry.

Parvoviruses are characterized by small‐sized nonenveloped single‐stranded DNA viruses.^1^ Human parvovirus B19 (B19V) and human parvovirus 4 (PARV4), members of family *Parvoviridae*, can infect humans and can be transmitted through blood components and blood products. B19V is much concerned in the fields of blood products because (1) it can present at extremely high titers in plasma of acutely infected but asymptomatic donors, thereby resulting in the highly contaminated source plasma pools for fractionation, (2) it is highly resistant to virus inactivation or removal methods used in the manufacture of blood products, and (3) sometimes it can cause severe diseases in at‐risk recipients.^2^ To mitigate the risk of B19V transmission, most manufacturers have been performing nucleic acid testing (NAT) for B19V DNA as an in‐process test for source plasma pools used for manufacturing certain or all kinds of blood products to limit the virus load, according to the guidance or standard from European Pharmacopoeia, the Plasma Protein Therapeutics Association, and U.S. Food and Drug Administration.^3^, ^4^, ^5^

Similar to B19V, PARV4 is also a frequent contaminant of source plasma pools for the production of blood products and the final products.^6^ Baylis et al.^7^ demonstrated PARV4 was even more resistant than B19V to virus inactivation strategies used during the manufacture of blood products, such as pasteurization and low‐pH treatment. The transmission of PARV4 by virally inactivated clotting factor concentrates raised concerns among the patients with hemophilia or other recipients of such products.^6^, ^8^ Given the clinical significance of PARV4 infection has not yet been confirmed, unlike B19V, there are no industry guidelines for restricting the level of PARV4 in source plasma pools. Despite the lack of clear evidence for PARV4‐mediated diseases, a variety of potential clinical associations have been proposed, including encephalitis, early human immunodeficiency virus (HIV)‐related symptoms, and fetal hydrops and hepatitis.^8^, ^9^, ^10^, ^11^, ^12^, ^13^ Moreover, Prakash et al.^14^ recently reported a strong association of PARV4 with severe respiratory illness. Same as B19V, PARV4 has been classified into three genotypes.^15^ All these three genotypes have been detected in human blood or blood products although they seem to have different epidemiology. Genotypes 1 and 2 are prevalent in North America, Europe, and some countries in Asia while genotype 3 seems to be endemic in Ghana.^16^ However, data on the existence of different PARV4 genotypes circulating in Chinese plasma donors are limited.

In our previous article, the prevalence of PARV4 in Chinese plasma pools has been reported, but the genotypes of PARV4 were not identified.^17^ The main objective of this study is to identify the genotypes of PARV4 circulating in Chinese plasma donors.

## 2 MATERIALS AND METHODS

### 2.1 Source plasma pool samples

A total of 78 source plasma pool samples (each comprising 2000–3000 donations) from one Chinese blood product manufacturer were analyzed in this study. All of such plasma samples were sourced from plasma donors in Central China and collected between April 2017 and June 2018. The collections of plasma samples were approved by the National Health Commission of the People's Republic of China, and all of the plasma donors provided informed consent. Every single plasma donation has gotten tested for infectious agents before and after the pooling and was confirmed to be qualified, according to the requirements of Pharmacopeia of the People's Republic of China.^18^

### 2.2 DNA isolation

Viral DNA was isolated from a volume of 200 μl of each plasma sample with the High Pure Viral Nucleic Acid Kit (Roche Diagnostics) according to the manufacturer's instructions. The concentration and purity of extracted DNA were measured using the GeneQuant 1300 spectrophotometer (GE Healthcare Bio‐sciences AB).

### 2.3 B19V and PARV4 quantitative polymerase chain reaction assay

The DNA samples were initially tested for presence and quantities of B19V and PARV4 DNA using a duplex quantitative polymerase chain reaction (qPCR) assay, which has been proved to be able to simultaneously detect and quantify all the known genotypes of B19V and PARV4, with an equal limit of quantification of 5 copies/ml.^19^ Serial log 10 dilutions of the B19V and PARV4 standard plasmids containing the qPCR target sequences and the samples were all analyzed in triplicate. On the basis of the standard curve generated by the standard plasmids and the quantification cycle value of each sample, the concentration of each virus DNA in copies/ml was calculated.

### 2.4 PARV4 nested PCR and sequencing

A 161‐bp region related to the NS1 gene of PARV4 (positions 1564–1724 in AY622943) was amplified by nested PCR with the primers described previously.^20^ PCR amplification products derived from each sample were purified and cloned into the pMD18‐T vector (TaKaRa Bio) and subsequently sequenced on an ABI 3730XL DNA Analyzer.

### 2.5 Phylogenetic analysis of PARV4 sequences

The PARV4 genotypes were determined by phylogenetic tree analysis based on the 161‐nt NS1 region (positions 1564–1724 in AY622943) using MEGA version 6.0. Twenty‐four PARV4 nonredundant sequences spanning this region were downloaded from GenBank (May 2020) and worked as the reference sequences. As reported previously, genetic distances of the sequences were calculated using the Kimura two‐parameter method, and phylogenetic trees were constructed by the neighbor‐joining method with 1000 bootstrap replicates.^21^

### 2.6 Nucleotide sequence accession numbers

The nucleotide sequences of the partial NS1 gene of the PARV4 have been lodged with the GenBank sequence database under the accession numbers MT873797 and MT873798.

## 3 RESULTS

### 3.1 Prevalence of B19V and PARV4 DNA in source plasma pools

Of 78 source plasma pool samples tested, 20 (25.64%) were positive for B19V DNA, 11 (14.10%) were positive for PARV4 DNA, only 1 (1.28%) was identified positive for both viral DNA, and 48 (61.54%) had no detectable levels of B19V and PARV4 nucleic acid.

### 3.2 Quantity of B19V and PARV4 DNA in source plasma pools

**Figure 1:**
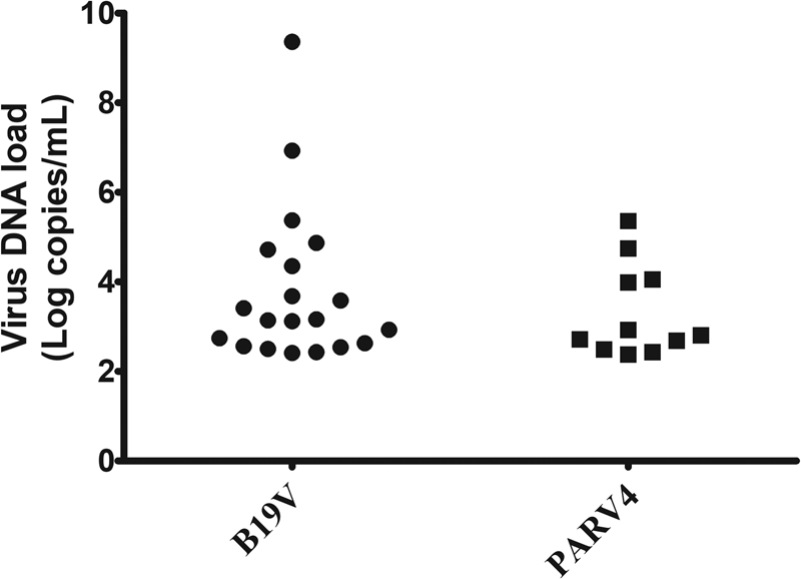
Distribution of the B19V and PARV4 DNA load in virus DNA‐positive source plasma pools. B19V, human parvovirus B19; PARV4, human parvovirus 4

The levels of B19V and PARV4 DNA in source plasma pools were shown in Figure 1. The quantity of B19V DNA varied from 2.56 × 10^2^ to 2.30 × 10^9^ copies/ml plasma. Levels of B19V DNA was as high as 2.30 × 10^9^ copies/ml plasma, although 70% of the positive samples were at low levels (10^2^–10^4^ copies/ml plasma). For PARV4, the level of virus DNA was lower than that of B19V: viral loads ranged from 2.42 × 10^2^ to 2.28 × 10^5^ copies/ml plasma, and most samples contained 10^2^–10^3^ copies/ml plasma. In the one coexistence sample, levels of B19V and PARV4 were low, equal to 1.31 × 10^3^ copies/ml plasma and 2.42 × 10^2^ copies/ml plasma, respectively.

### 3.3 Genotypes of PARV4 sequences in source plasma pools

The genotypes of PARV4 sequences in source plasma pool samples were identified. Of the 11 samples found to be positive for PARV4 sequences by qPCR (Table 1), only 2 were positive for PARV4 nested PCR and available for further analysis. The PARV4 DNA quantity of these two samples was 5.28 × 10^2^ and 9.75 × 10^3^ copies/ml plasma, respectively. While no targeted PCR products were amplified from five samples containing greater than 5.28 × 10^2^ copies/ml plasma of PARV4 DNA. These unexpected results might attribute to the incomplete sequence of PARV4 genome in these samples, in view of the different target regions between PARV4 qPCR and nested PCR.

**Table 1:** Details of plasma pools containing PARV4

Sample No.	B19V (copies/ml)	PARV4 (copies/ml)	Genotype of PARV4
3	Neg	4.95 × 10^2^	–
7	Neg	5.28 × 10^2^	G1
10	Neg	2.70 × 10^2^	–
11	Neg	2.28 × 10^5^	–
13	Neg	3.12 × 10^2^	–
14	Neg	9.75 × 10^3^	G2
15	Neg	1.14 × 10^4^	–
21	Neg	5.59 × 10^4^	–
32	Neg	8.57 × 10^2^	–
35	1.31 × 10^3^	2.42 × 10^2^	–
36	Neg	6.47 × 10^2^	–

*Note*: “–” indicates that the genotype of PARV4 in the sample was not identified.

Abbreviations: B19V, human parvovirus B19; PARV4, human parvovirus 4; Neg, negative.

**Figure 2:**
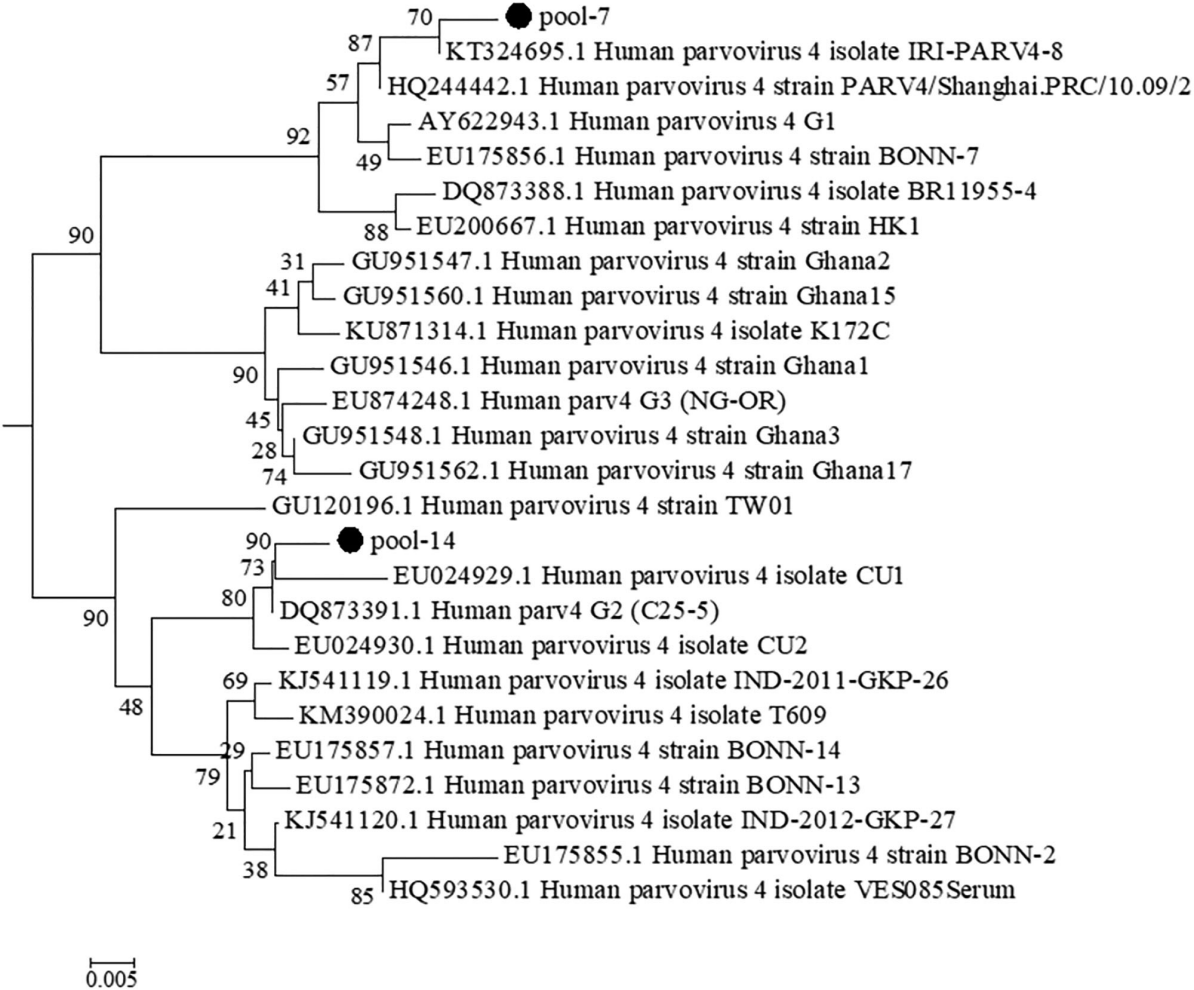
Phylogenetic analysis of human parvovirus 4 (PARV4) nucleotide sequences. The phylogenetic tree was constructed based on the 161‐nt NS1 region of PARV4 and the neighbor‐joining algorithm using the Kimura two‐parameter model. Two PARV4 sequences from this study (labeled with black circles) and a set of PARV4 sequences downloaded from GenBank (labeled with their GenBank accession number and isolate or strain name) used as references for the different genotypes were analyzed. Bootstrap replication frequencies are indicated above each node. Branch lengths are drawn to scale

Phylogenetic analysis of 2 sequences obtained in this study, together with 24 sequences retrieved from GenBank, revealed that one sequence (pool‐7) clustering together with genotype‐1 reference sequences belonged to PARV4 genotype 1, and the other one sequence (pool‐14) clustering together with genotype‐2 reference sequences belonged to PARV4 genotype 2 (Figure 2).

## 4 DISCUSSION

Since discovered in 2005, PARV4 has received much attention.^22^ Unlike B19V, which took nearly 30 years after its initial discovery in 1975 to identify its three genotypes, PARV4 genotypes 2 and 3 were identified within 3 years.^15^, ^23^, ^24^, ^25^ Research on the prevalence and distribution of different genotypes contributes to our understanding of human parvovirus evolution and diversification and enables good assay design for virus detection. Accumulating studies have reported the PARV4 genotypes prevalent in blood or plasma donors of many countries, however, no relevant data have ever been reported in China.^20^, ^26^, ^27^

This is the first report to identify the genotypes of PARV4 circulating in Chinese plasma donors. Source plasma pools are ideal materials for blood‐borne virus genotype studies. In our previous studies, the source plasma pools were used as the samples for B19V genotyping, and first reported the cocirculation of B19V 1a, B19V 1b, and B19V 3b, as well as the putative B19V 1/3 recombinant and new strains in Chinese plasma donations.^21^ In this study, the PARV4 sequences in two PARV4‐DNA positive source plasma pool samples were genotyped and the results demonstrated that at least two PARV4 genotypes, 1 and 2, were currently present in China. No genotype 3 was detected, which was not unexpected since this genotype was reported to be endemic in Ghana.^16^ Besides, the possibility that genotype 3 might be present at an extremely low level in the sample, and, therefore, escaped identification can not be completely ruled out. Furthermore, it should be noted that this study was restricted to one region of China, and, therefore, could not reflect the whole national circulating status of different PARV4 genotypes. B19V genotype 3 is originally endemic to Ghana as well, but as research continues it shows a wider distribution and has been found in Brazil, France, North India, the United States, and China.^21^, ^28^ Whether PARV4 genotype 3 will show an increasing spread also outside of Ghana, just like B19V, needs further research with large sample size and a wide geographical area.

These results also showed that the positive rates and levels of B19V DNA in Chinese plasma pool samples were relatively higher than that of PARV4 DNA, indicating that the prevalence of B19V DNA in the Chinese population might be higher than that of PARV4, consistent with results detected in the general population from a previous report.^29^ On the other hand, regarding the frequent coinfection with hepatitis C virus and HIV, some PARV4 positive plasma samples might be excluded by infectious agents screening tests before pooling.^6^ The prevalence of B19V DNA in source plasma pool samples tested in this study was lower than that reported in 2015 (104/141, 73.76%), whose samples derived from the same manufacture were collected between 2008 and 2013.^30^ Given the duplex qPCR assay used in this study has high sensitivity, the diversity can be mainly attributed to the differences in the year and season of sample collection. Besides, differences in sample size and the number of plasma units within each pooled sample might be the influence factors on such diversity. Also, the prevalence of B19V DNA tested in this manufacturer was lower than that in other manufacturers in China (5.45%–100%) as well as other countries before NAT was introduced (56.10%–59.68%), reflecting the geographic and temporal differences in the prevalence of the virus, differences in detection methods, as well as the differences in the number of plasma units within each pooled sample and in the sample size.^31^ For PARV4, the prevalence in samples collected between 2017 and 2018 in this study (11/78, 14.10%) was lower than collected between 2007 and 2010 from the same manufacture (39/101, 38.61%), indicating the seasonal epidemic variation, whereas was equivalent to other two manufacturers (3/20, 15% and 9/74, 12.16%, respectively) in China.^17^

In our study, out of the 30 parvovirus DNA‐positive source plasma pool samples, only one was identified positive for both B19V and PARV4 DNA. The low rate of coinfection indicated that the PARV4 was not associated with the infection of B19V. Thus, the implementation of B19V NAT assays was not able to reduce the possible risk of PARV4 transmission by blood products. The transfusion‐mediated transmission of PARV4 remains a concern. Given that associated disease or specific symptoms have not yet been confirmed, there is no need to exclude PARV4 from source plasma pools, at least for the time being.^6^

It should be noted that although the use of source plasma pools as materials in this study increases the potential for the detection of PARV4, the data regarding PARV4 containing plasma pools were still relatively limited. In further research, screen and sequence more source plasma pool samples by extracting larger volumes of plasma or concentrating virions by immune adsorption could address this limitation.

## 5 CONCLUSION

In conclusion, the data present demonstrate the existence of PARV4 genotype 1 and 2 in Chinese plasma donors for the first time and also show the relatively lower prevalence and level of PARV4 DNA in Chinese plasma donors in comparison with that of B19V DNA.

## CONFLICT OF INTERESTS

The authors declare that there are no conflict of interests.

## AUTHOR CONTRIBUTIONS

Junting Jia searched data and drafted the manuscript. Junting Jia and Yadi Zhong performed the experiments and analyzed the data. Yuyuan Ma, Jingang Zhang, and Deqing Wang designed the study and provided critical revision of the article. Huan Zhang, Dian Yuan, and Limin Ma provided help during the process of experiments. All authors reviewed and approved the final manuscript.

## Data Availability

Data are available on request from the authors. The data that support the findings of this study are available from the corresponding author upon reasonable request.

## References

[1] Cotmore SF, Agbandje‐McKenna M, Canuti M, et al. ICTV virus taxonomy profile: *Parvoviridae* . J Gen Virol. 2019;100(3):367‐368. 30672729 10.1099/jgv.0.001212 PMC6537627

[2] Marano G, Vaglio S, Pupella S, et al. Human parvovirus B19 and blood product safety: a tale of twenty years of improvements. Blood Transfus. 2015;13(2):184‐196. 25849894 10.2450/2014.0174.14 PMC4385066

[3] Plasma Protein Therapeutics Association . *QSEAL NAT testing standard (version 2.0)*; 2013. http://www.pptaglobal.org/images/qseal/NATTestingV2.pdf. Accessed August 11, 2020.

[4] U.S. Department of Health and Human Services, Food and Drug Administration, Center for Biologics Evaluation and Research . *Guidance for industry: nucleic acid testing (NAT) to reduce the possible risk of parvovirus B19 transmission by plasma‐derived products*; July 2009. https://www.fda.gov/regulatory-information/search-fda-guidance-documents/nucleic-acid-testing-reduce-possible-risk-parvovirus-b19-transmission-plasma-derived-products. Accessed August 11, 2020.

[5] Council of Europe. European Pharmacopoeia. 9th ed. Directorate for the Quality of Medicines of the Council of Europe (EDQM); 2017.

[6] Delwart E. Human parvovirus 4 in the blood supply and transmission by pooled plasma‐derived clotting factors: does it matter? Transfusion. 2012;52(7):1398‐1403. 22780892 10.1111/j.1537-2995.2012.03721.x PMC3666916

[7] Baylis SA, Tuke PW, Miyagawa E, Blumel J. Studies on the inactivation of human parvovirus 4. Transfusion. 2013;53(10 Pt 2):2585‐2592. 24032592 10.1111/trf.12372

[8] Sharp CP, Lail A, Donfield S, Gomperts ED, Simmonds P. Virologic and clinical features of primary infection with human parvovirus 4 in subjects with hemophilia: frequent transmission by virally inactivated clotting factor concentrates. Transfusion. 2012;52(7):1482‐1489. 22043925 10.1111/j.1537-2995.2011.03420.x

[9] Benjamin L., Human parvovirus 4 as potential cause of encephalitis in children, India. Emerg Infect Dis. 2011;17(8):1484‐1487. 21801629 10.3201/eid1708.110165 PMC3381555

[10] Prakash S, Jain A, Seth A, Singh AK, Jain B. Complete genome sequences of two isolates of human parvovirus 4 from patients with acute encephalitis syndrome. Genome Announc. 2015;3(1):e01472‐14. 10.1128/genomeA.01472-14 PMC4319504 25635010

[11] Arankalle VA, Srivastava N, Kushwaha KP, et al. Detection of human parvovirus 4 DNA in the patients with acute encephalitis syndrome during seasonal outbreaks of the disease in Gorakhpur, India. Emerg Microbes Infect. 2019;8(1):130‐138. 30866767 10.1080/22221751.2018.1563455 PMC6455185

[12] Simmons R, Sharp C, McClure CP, et al. Parvovirus 4 infection and clinical outcome in high‐risk populations. J Infect Dis. 2012;205(12):1816‐1820. 22492853 10.1093/infdis/jis291 PMC3357136

[13] Chen MY, Yang SJ, Hung CC. Placental transmission of human parvovirus 4 in newborns with hydrops, Taiwan. Emerg Infect Dis. 2011;17(10):1954‐1956. 22000381 10.3201/eid1710.101841 PMC3310659

[14] Prakash S, Shukla S, Ramakrishna V, Mishra H, Bhagat AK, Jain A. Human parvovirus 4: a harmless bystander or a pathogen of severe acute respiratory illness. Int J Infect Dis. 2020;90:21‐25. 31605808 10.1016/j.ijid.2019.10.001 PMC7172059

[15] Simmonds P, Douglas J, Bestetti G, et al. A third genotype of the human parvovirus PARV4 in sub‐Saharan Africa. J Gen Virol. 2008;89(Pt 9):2299‐2302. 18753240 10.1099/vir.0.2008/001180-0

[16] Soderlund‐Venermo M. Emerging human parvoviruses: the rocky road to fame. Annu Rev Virol. 2019;6(1):71‐91. 31283445 10.1146/annurev-virology-092818-015803

[17] Ma YY, Guo Y, Zhao X, et al. Human parvovirus PARV4 in plasma pools of Chinese origin. Vox Sang. 2012;103(3):183‐185. 22458565 10.1111/j.1423-0410.2012.01605.x

[18] Chinese Pharmacopoeia Commission. Human plasma for manufacturing plasma derivatives. The Pharmacopoeia of the People's Republic of China. Vol 3. China Medical Science Press; 2015:16‐18.

[19] Jia J, Zhong Y, Guo Y, et al. Simultaneous detection and differentiation of human parvovirus B19 and human parvovirus 4 by an internally controlled multiplex quantitative real‐time PCR. Mol Cell Probes. 2017;36:50‐57. 28863892 10.1016/j.mcp.2017.08.005

[20] Asiyabi S, Nejati A, Shoja Z, et al. First report of human parvovirus 4 detection in Iran. J Med Virol. 2016;88(8):1314‐1318. 26812938 10.1002/jmv.24485

[21] Jia J, Ma Y, Zhao X, et al. Existence of various human parvovirus B19 genotypes in Chinese plasma pools: genotype 1, genotype 3, putative intergenotypic recombinant variants and new genotypes. Virol J. 2016;13(1):155. 27639978 10.1186/s12985-016-0611-6 PMC5027099

[22] Jones MS, Kapoor A, Lukashov VV, Simmonds P, Hecht F, Delwart E. New DNA viruses identified in patients with acute viral infection syndrome. J Virol. 2005;79(13):8230‐8236. 15956568 10.1128/JVI.79.13.8230-8236.2005 PMC1143717

[23] Cossart YE, Field AM, Cant B, Widdows D. Parvovirus‐like particles in human sera. Lancet. 1975;1(7898):72‐73. 46024 10.1016/s0140-6736(75)91074-0

[24] Servant A, Laperche S, Lallemand F, et al. Genetic diversity within human erythroviruses: identification of three genotypes. J Virol. 2002;76(18):9124‐9134. 12186896 10.1128/JVI.76.18.9124-9134.2002 PMC136440

[25] Fryer JF, Delwart E, Bernardin F, Tuke PW, Lukashov VV, Baylis SA. Analysis of two human parvovirus PARV4 genotypes identified in human plasma for fractionation. J Gen Virol. 2007;88(Pt 8):2162‐2167. 17622618 10.1099/vir.0.82620-0

[26] Fryer JF, Kapoor A, Minor PD, Delwart E, Baylis SA. Novel parvovirus and related variant in human plasma. Emerg Infect Dis. 2006;12(1):151‐154. 16494735 10.3201/eid1201.050916 PMC3291395

[27] Lurcharchaiwong W, Chieochansin T, Payungporn S, Theamboonlers A, Poovorawan Y. Parvovirus 4 (PARV4) in serum of intravenous drug users and blood donors. Infection. 2008;36(5):488‐491. 18759058 10.1007/s15010-008-7336-4

[28] Hubschen JM, Mihneva Z, Mentis AF, et al. Phylogenetic analysis of human parvovirus b19 sequences from eleven different countries confirms the predominance of genotype 1 and suggests the spread of genotype 3b. J Clin Microbiol. 2009;47(11):3735‐3738. 19741071 10.1128/JCM.01201-09 PMC2772644

[29] Tong R, Shen L, Yin W, et al. Prevalence of human parvovirus B19, bocavirus, and PARV4 in blood samples from the general population of China and lack of a correlation between parvovirus and hepatitis B co‐infection. PLoS One. 2013;8(5):e64391. 23737981 10.1371/journal.pone.0064391 PMC3667789

[30] Jia J, Ma Y, Zhao X, et al. Prevalence of human parvovirus B19 in Chinese plasma pools for manufacturing plasma derivatives. Virol J. 2015;12:162. 26445095 10.1186/s12985-015-0396-z PMC4596515

[31] Jia J, Zhang M, Ma Y, Zhang J. Human parvovirus B19 research concerning the safety of blood and plasma derivatives in China. Ann Blood. 2019;4:2.

